# Application of CT and MRI images based on artificial intelligence to predict lymph node metastases in patients with oral squamous cell carcinoma: a subgroup meta-analysis

**DOI:** 10.3389/fonc.2024.1395159

**Published:** 2024-06-18

**Authors:** Cheng Deng, Jun Hu, Ping Tang, Tao Xu, Ling He, Zesheng Zeng, Jianfeng Sheng

**Affiliations:** Department of Thyroid, Head, Neck and Maxillofacial Surgery, the Third Hospital of Mianyang & Sichuan Mental Health Center, Mianyang, Sichuan, China

**Keywords:** oral squamous cell carcinoma, artificial intelligence, radiomics, deep learning, lymph node metastasis, meta-analysis

## Abstract

**Background:**

The performance of artificial intelligence (AI) in the prediction of lymph node (LN) metastasis in patients with oral squamous cell carcinoma (OSCC) has not been quantitatively evaluated. The purpose of this study was to conduct a systematic review and meta-analysis of published data on the diagnostic performance of CT and MRI based on AI algorithms for predicting LN metastases in patients with OSCC.

**Methods:**

We searched the Embase, PubMed (Medline), Web of Science, and Cochrane databases for studies on the use of AI in predicting LN metastasis in OSCC. Binary diagnostic accuracy data were extracted to obtain the outcomes of interest, namely, the area under the curve (AUC), sensitivity, and specificity, and compared the diagnostic performance of AI with that of radiologists. Subgroup analyses were performed with regard to different types of AI algorithms and imaging modalities.

**Results:**

Fourteen eligible studies were included in the meta-analysis. The AUC, sensitivity, and specificity of the AI models for the diagnosis of LN metastases were 0.92 (95% CI 0.89–0.94), 0.79 (95% CI 0.72–0.85), and 0.90 (95% CI 0.86–0.93), respectively. Promising diagnostic performance was observed in the subgroup analyses based on algorithm types [machine learning (ML) or deep learning (DL)] and imaging modalities (CT vs. MRI). The pooled diagnostic performance of AI was significantly better than that of experienced radiologists.

**Discussion:**

In conclusion, AI based on CT and MRI imaging has good diagnostic accuracy in predicting LN metastasis in patients with OSCC and thus has the potential for clinical application.

**Systematic Review Registration:**

https://www.crd.york.ac.uk/PROSPERO/#recordDetails, PROSPERO (No. CRD42024506159).

## 1 Introduction

Oral squamous cell carcinoma (OSCC) is the most common type of oral cancer, accounting for approximately 90% of oral cancer cases and with a poor prognosis (1, 2). Assessing the lymph node (LN) status determines the staging, management, and ultimately the survival outcomes of patients with OSCC (3–5). Cervical LN metastasis is associated with poor prognosis and is one of the most important independent prognostic factors in OSCC (6, 7). The dissection of metastatic LNs at the time of resection of the primary tumor can significantly reduce the rate of regional recurrence and enhance the survival of patients with OSCC (8, 9). Thus, the accurate determination of the clinical LN status is critical for the treatment and prognosis of these patients. Computed tomography (CT) and magnetic resonance imaging (MRI) are widely used to evaluate the status of cervical LNs in patients with OSCC (10, 11). However, the diagnostic accuracy of these methods is affected by multiple factors and remains inadequate for assessing the LN status in these patients (12, 13).

Artificial intelligence (AI) has recently been applied to the evaluation of radiology images, as it excels at automatically recognizing complex patterns in imaging data and providing quantitative, rather than qualitative, assessments of radiographic characteristics (14). Currently, radiological feature-based AI plays an important role in tumor diagnosis and staging, as well as in predicting the treatment response and prognosis, demonstrating its potential as a non-invasive auxiliary tool for personalized medicine (15, 16). In recent years, several studies have reported the application of AI algorithms [machine learning (ML) or deep learning (DL)] in predicting LN metastasis in OSCC patients, and they exhibited promising performance. However, available information on the use of AI-based methods for the prediction of OSCC LN status is scattered, and thus, a systematic review to evaluate and summarize the prediction performance of AI-based methods using CT and MRI is needed.

Thus, the purpose of this study was to conduct a systematic review and meta-analysis of published data on the performance of CT and MRI based on AI algorithms for predicting LN metastases in patients with OSCC. In addition, to elaborate on the predictive performance of AI and increase the reliability of the evidence, a subgroup meta-analysis was performed by grouping studies according to the type of AI algorithm and image modality.

## 2 Methods

A systematic search of the Cochrane Library, PubMed (Medline), Embase, and Web of Science was performed using Preferred Reporting Items for Systematic Reviews and Meta-analysis (PRISMA) guidelines (17). This study was prospectively registered in PROSPERO (No. CRD42024506159).

### 2.1 Search strategy

All potentially relevant studies from the beginning up to February 2024 were identified. The following MeSH terms were used: “oral squamous cell carcinoma,” “oral tongue squamous cell carcinoma,” “head and neck squamous cell carcinoma,” “artificial intelligence,” “deep learning,” “convolutional neural network,” “machine learning,” “automatic detection,” “radiomics,” “radiomic,” “CT”, “MRI,” “lymph node,” and “lymph node metastasis.” The details of the search formula are shown in **Supplementary Table 1**: “Search strategy.” Additional studies were identified by hand-searching reference lists of all relevant articles. Any disagreements in the results of the search process between two authors were resolved by a discussion or consultation with a third author (the corresponding author).

### 2.2 Selection criteria

Articles were included if they met the following criteria: 1) included patients with histopathological diagnosis of OSCC; 2) developed or used ML or DL to assess CT and MRI preoperative lymph node metastasis prediction; 3) can estimate the values of true-positive (TP), false-positive (FP), false-negative (FN), and true-negative (TN); and 4) published in the English language. The exclusion criteria were as follows: 1) studies with valid outcomes data that could not be extracted; 2) reviews, guidelines, and meta-analyses; 3) animal experiments, case reports, abstracts, conference proceedings, or expert opinions; and 4) duplicate publications.

### 2.3 Data extraction

Two authors independently extracted the data from each included study and cross-checked the extracted data. If the data were unclear, the corresponding author of the study was contacted by email to obtain insight into the original data set. Any disagreements in the results of the search process between the two authors were resolved by a discussion or consultation with a third author (the corresponding author).

The following data were then extracted from each study: study type, area of the study, total patient number, sample size for diagnostic accuracy, targeted area, image modality, AI algorithm, and reference gold standard. To obtain diagnostic accuracy data, we extracted TP, FP, TN, FN, and area under the receiver operating curve (AUC) along with other parameters of the AI models. If the included studies presented comparative diagnostic performance of AI models versus radiologists, the TP, FP, FN, and TN values of the radiologists in each study were also extracted.

For subgroup analysis, the authors extracted the following variables from each included study: type of AI algorithms (ML or DL) and type of image modality (CT or MRI).

### 2.4 Quality assessment

Two authors assessed the methodological quality of the final included articles using the second version of the Quality Assessment of Diagnostic Accuracy Studies (QUADAS-2). Two authors assessed each part as having a high, low, or unclear risk of bias.

### 2.5 Publication bias

Publication bias was evaluated using the Deek funnel plot asymmetry test.

### 2.6 Statistical analysis

For the quantitative meta-analysis, TP, FP, TN, and FN extracted from the test set were used. If results were not reported in an independent test set, cross-validation results are reported. When different AI models were tested within the same paper, the proposed model in the paper with the highest diagnostic performance was used for pooled analysis and the needed model was used for subgroup analysis, respectively. Sensitivity analyses were carried out by sequentially removing individual studies to evaluate the robustness of the pooled results. Additionally, a pooled analysis was performed to estimate the accuracy of the radiologist’s assessment derived from studies that reported this. The corresponding AUC, sensitivities, and specificities of the radiologists were extracted in the same way as described above.

Statistical analyses were performed with STATA (version 15.1; Stata Corporation, College Station, TX, USA) software, including the packages metandi and midas. The bivariate method and the hierarchical summary receiver operating characteristic (HSROC) method were applied for meta-analysis (18, 19). A forest plot of the sensitivity and specificity and a summary receiver-operating characteristic (SROC) curve were generated using the bivariate method and HSROC method, respectively. To assess heterogeneity between studies, the inconsistency index (*I* ^2^) was used. *I* ^2^ values below 50% indicated low heterogeneity, while values above 50% indicated substantial heterogeneity (20). All tests were two-tailed tests and a difference of *P <*0.05 was considered statistically significant. Differences between subgroups were assessed by inspection of the subgroups’ confidence intervals. Non-overlapping confidence intervals for any two subgroups indicated a statistically significant difference between the subgroups (21, 22).

## 3 Results

### 3.1 Study selection

**Figure 1:**
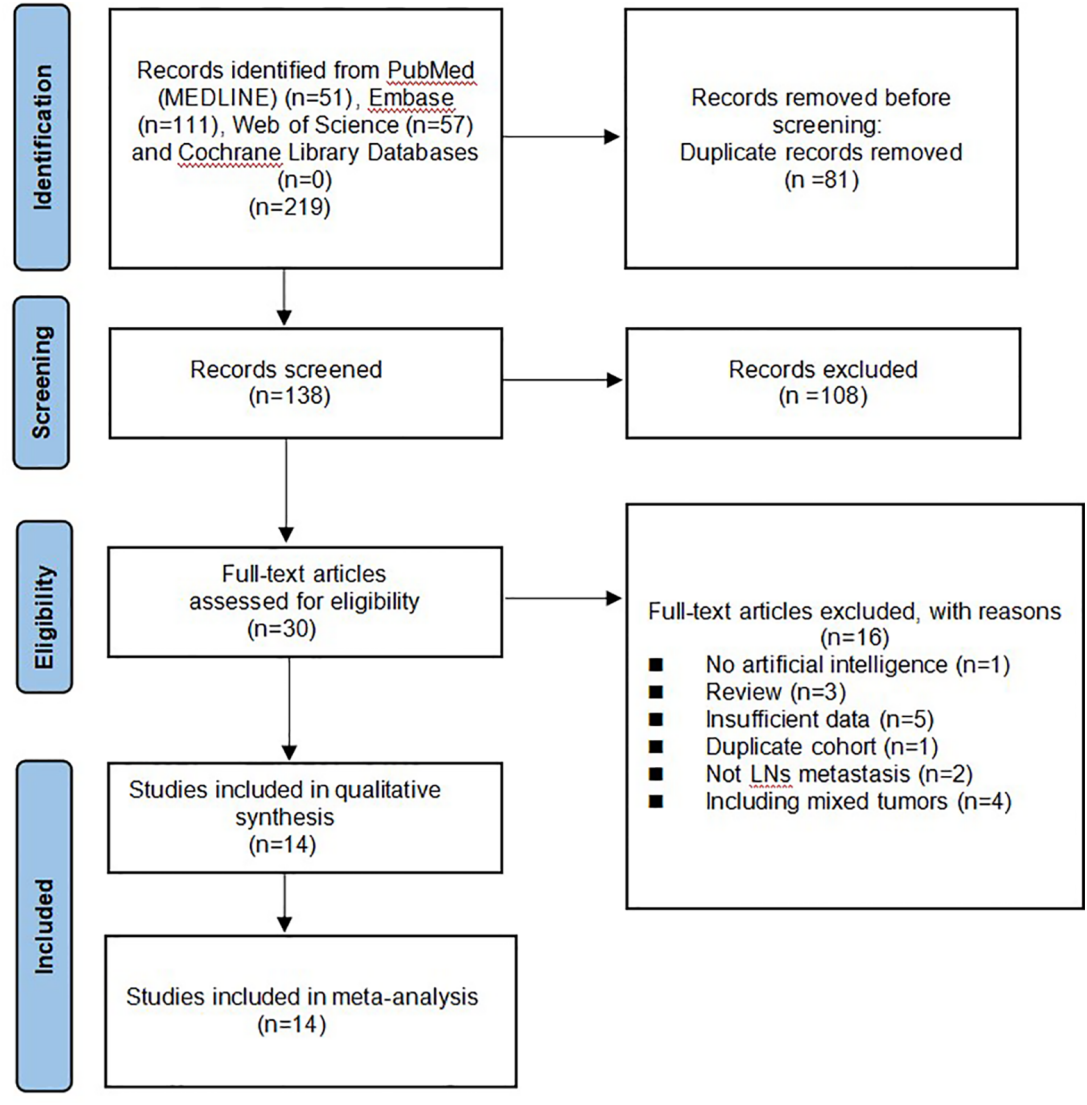
Steps of the process of screening documents.

A total of 219 studies were identified and 138 remained after removing duplicates. A review of the titles and abstracts left 28 studies for full-text review. Finally, 14 (19–32) articles were included in the systematic review and meta-analysis. The flowchart of the search and screening results for the relevant studies is shown in **Figure 1**.

### 3.2 Study characteristics

The characteristics of the included studies are summarized in **Table 1**. The included studies were published between 2019 and 2023 and all of them were retrospective. Seven studies were performed in China and the rest were performed in Japan (five studies) and Italy (two studies). In terms of AI algorithms, five studies used the DL algorithm and 10 studies used the ML algorithm (one of them used both AI algorithms and separately evaluated the performance of the fusion model). For radiomic features, LNs or tumor features in MRI were extracted by five studies and features in CT were extracted by nine studies. Regarding the extracting method, only two studies automatically extracted the feature, while others extracted the feature manually by experienced radiologists. Seven studies presented the comparative diagnostic performance of AI models versus experienced radiologists.

**Table 1:** Characteristics of the included studies.

Study, year	Study type	country	No. of patients	Year of recruitment	Image modality	Method	Targeted area	Segmentation	TP	FP	FN	TN	Sample size for diagnostic accuracy, *n* (dataset)	Reference standard	Radiologists control
Ariji, 2019 (23)	Retrospective	Japan	45	2007–2015	CT	Deep learning	LN	Manual	103	77	24	237	441 (cross-validation)	Pathology	Yes
Tomita (1), 2021 (24)	Retrospective	Japan	23	2013–2017	CT	Machine learning	LN	Manual	16	2	4	38	60 (validation)	Pathology	Yes
Tomita (2), 2021 (25)	Retrospective	Japan	39	2013–2017	CT	Deep learning	LN	Manual	14	2	7	41	64 (testing)	Pathology	Yes
Yuan, 2021 (26)	Retrospective	China	116	2015–2019	MRI	Machine learning	Tumor	Manual	31	10	18	57	116 (cross-validation)	Pathology	No
Ariji, 2022 (27)	Retrospective	Japan	59	2007–2019	CT	Deep learning	Tumor	Automated	23	3	1	65	92 (testing)	Pathology	Yes
Committeri, 2022 (28)	Retrospective	Italy	81	2016–2020	CT	Machine learning	Tumor	Manual	31	5	0	45	81 (cross-validation)	Pathology	No
Kubo, 2022 (29)	Retrospective	Japan	161	2008–2019	CT	Machine learning	LN	Manual	38	16	8	99	161 (cross-validation)	Pathology	No
Ren, 2022 (30)	Retrospective	China	55	2015–2021	MRI	Machine learning	Tumor	Manual	27	3	7	18	55 (cross-validation)	Pathology	No
Wang, 2022 (31)	Retrospective	China	79	2012–2019	MRI	Machine learning	Tumor	Manual	26	4	7	42	79 (cross-validation)	Pathology	No
Zhong, 2022 (32)	Retrospective	China	313	2013–2018	CT	Machine learning	Tumor	Manual	17	1	10	35	63 (testing)	Pathology	No
Chen, 2023 (33)	Retrospective	China	100	2013–2016	CT	DL_ML^a^	LN	Manual	46	57	4	457	564 (testing)	Pathology	Yes
Liu, 2023 (34)	Retrospective	China	400	2013–2022	MRI	Machine learning	Tumor	Manual	17	5	8	28	58 (testing)	Pathology	Yes
Xu, 2023 (35)	Retrospective	China	1466	2012–2020	CT	Deep learning	Tumor	Automated	182	35	67	723	1,007 (testing)	Pathology	Yes
Vidiri, 2023 (36)	Retrospective	Italy	108	2013–2022	MRI	Machine learning	Tumor	Manual	9	6	6	15	36 (validation)	Pathology	No

a Deep learning and machine learning fusion model.

LN, lymph node; TP, true positive; FP, false positive; FN, false negative; TN, true negative.

### 3.3 Quality assessment and publication bias

**Figure 2:**
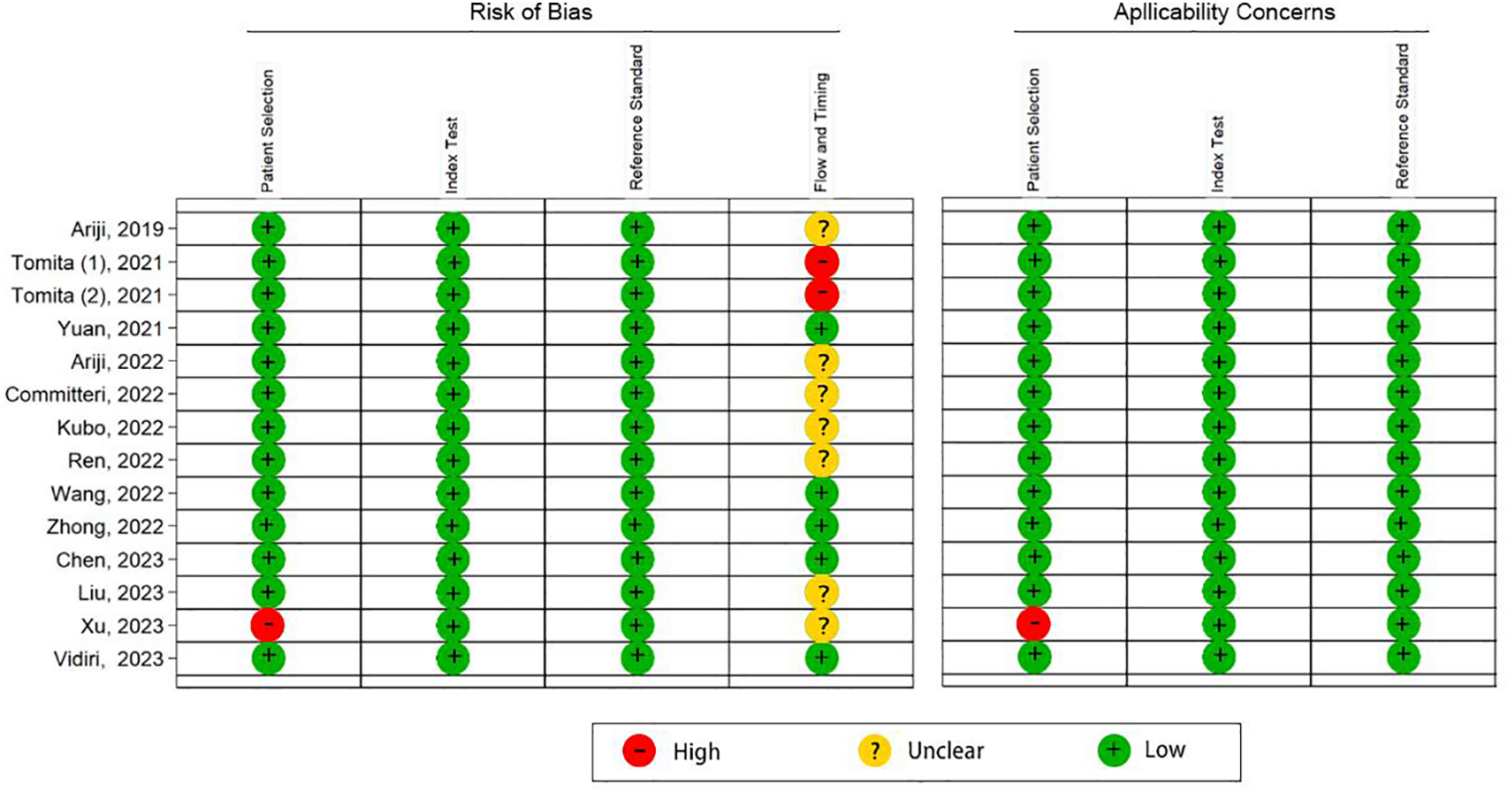
Quality assessment of the 14 included studies by the QUADAS-2 tool. “+” denotes a low risk of bias, “?” denotes an unclear risk of bias, and “−” denotes a high risk of bias.

**Figure 3:**
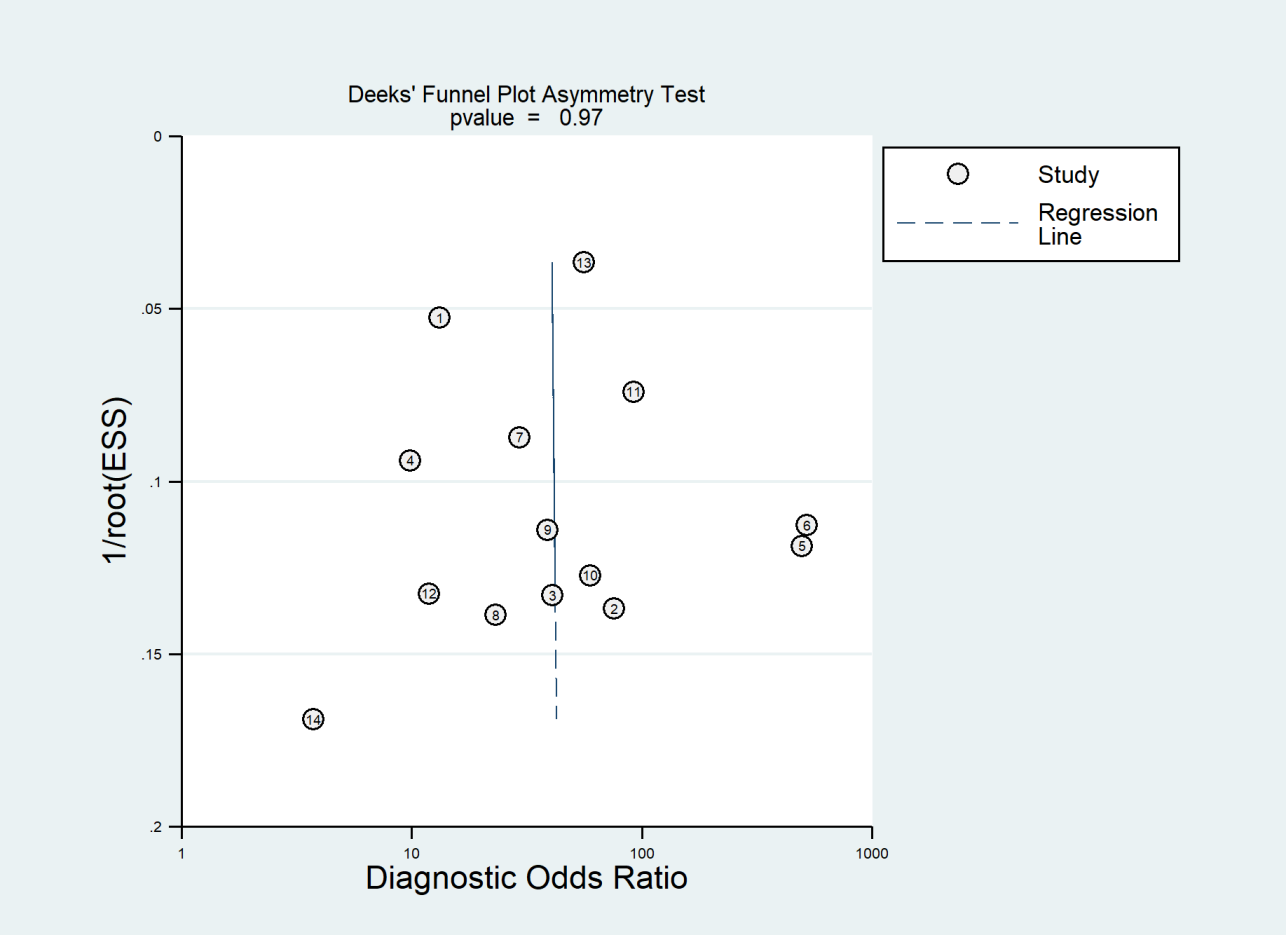
Deek funnel plot of artificial intelligence (AI) models for the prediction of lymph node (LN) metastasis.

According to the QUADAS-2 tool, the overall risk of bias in the selection of patients was high in one (7%) study and low in 13 (93%) studies. Flow and timing was assessed in only five (36%) studies with a low risk of bias. Overall applicability concerns were low except for one (7%) study with a high risk of bias in the patient selection. Individual evaluation of the risk of bias and its applicability is shown in **Figure 2**. The Deek funnel plot exhibited a symmetrical shape with respect to the regression line (**Figure 3**), and the asymmetry test showed no evidence of publication bias (*P* = 0.97). The detailed results of the quality assessment for each study are shown in **Supplementary Table 2**: “Quality assessment.”

### 3.4 Meta-analysis of diagnostic accuracy

**Figure 4:**
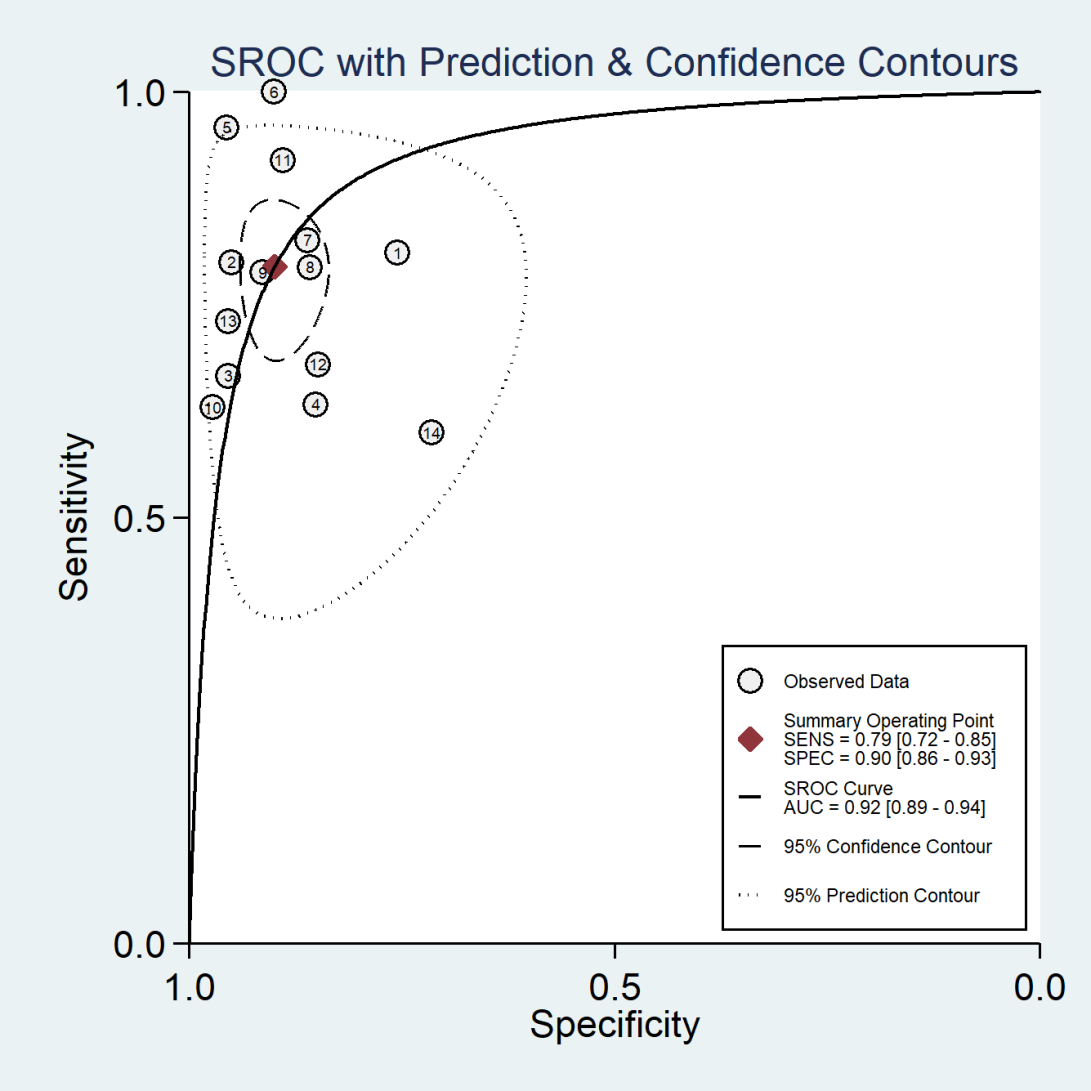
The summary receiver-operating characteristic (SROC) curves of AI models for the prediction of LN metastasis.

**Figure 5:**
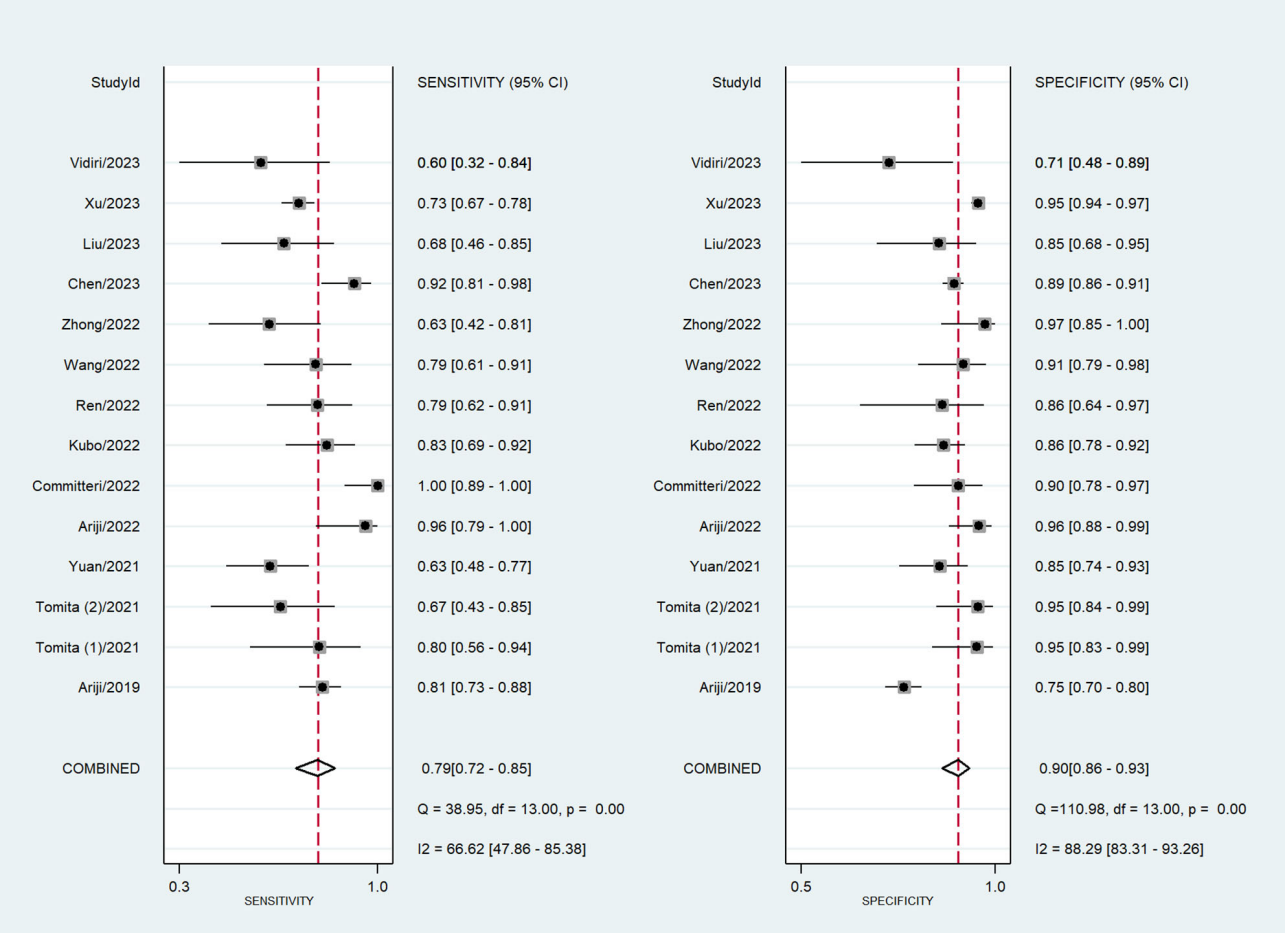
Coupled forest plots of sensitivity and specificity in AI models for the prediction of LN metastasis.

**Figure 6:**
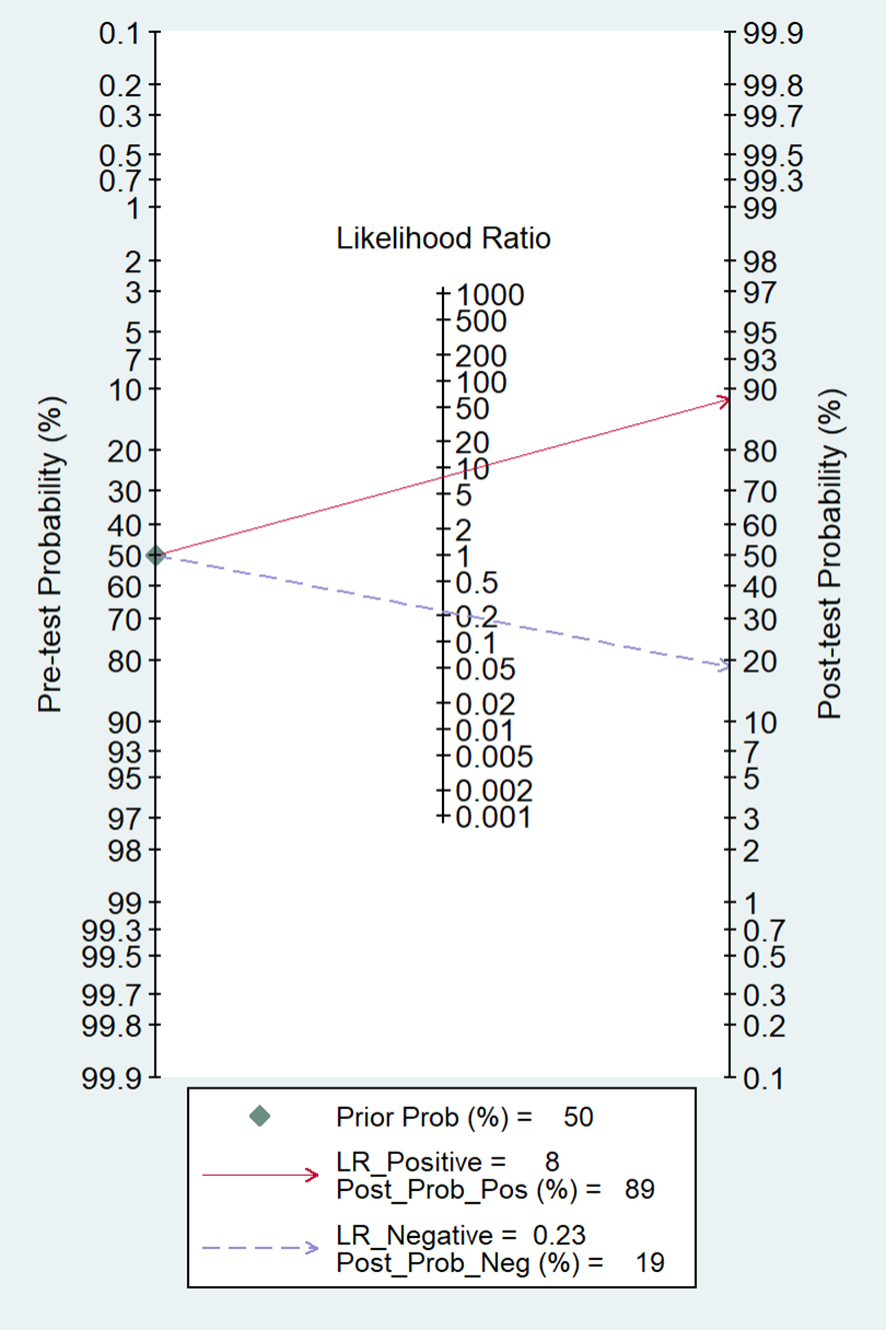
The Fagan nomogram for the prediction of LN metastasis. LR, likelihood ratio; Post_Prob_Pos, the posterior probability of LN metastasis if the finding of the model was positive; Post_Prob_Neg, the posterior probability of LN metastasis if the finding of the model was negative.

Among the 14 studies for the meta-analysis, the pooled AUC, sensitivity, specificity, positive likelihood ratio, negative likelihood ratio, and DOR of AI models for the diagnosis of LN metastases were 0.92 (95% CI 0.89–0.94), 0.79 (95% CI 0.72–0.85), 0.90 (95% CI 0.86–0.93), 7.9 (95% CI 5.5–11.4), 0.23 (95% CI 0.16–0.32), and 35 (95% CI 19–62), respectively (**Table 2**). The SROC curve with a 95% confidence region and prediction region is illustrated in **Figure 4**. The forest plots of sensitivity and specificity are illustrated in **Figure 5**. To investigate the clinical utility of the AI models, a Fagan nomogram was generated. Assuming a 50% prevalence of LN metastasis, the Fagan nomogram shows that the posterior probability of LN metastasis was 89% if the test was positive, and the posterior probability of LN metastasis was 19% if the test was negative (**Figure 6**). For sensitivity analyses, after each exclusion of a single study, there was no large variation in the results, suggesting the stability of the findings; however, high heterogeneity among the studies remains.

**Table 2:** Summary of results.

Subgroup	Included studies (*n* = 14)	AUC (95% CI)	Sensitivity (95% CI)	Specificity (95% CI)	PLR, mean (95% CI)	NLR, mean (95% CI)	DOR (95% CI)
Value of meta-analysis in all the included studies	14	0.92 (0.89–0.94)	0.79 (0.72–0.85)	0.90 (0.86–0.93)	7.9 (5.5–11.4)	0.23 (0.16–0.32)	35 (19–62)
Type of AI algorithms							
ML	10	0.91 (0.88–0.93)	0.84 (0.74–0.90)	0.87 (0.82–0.90)	6.3 (4.6–8.4)	0.18 (0.11–0.30)	34 (19–61)
DL	5	0.92 (0.89–0.94)	0.74 (0.44–0.91)	0.90 (0.81–0.95)	7.7 (4.0–14.8)	0.29 (0.11–0.72)	27 (8–92)
Type of image modality							
CT	9	0.94 (0.92–0.96)	0.81 (0.62–0.92)	0.92 (0.87–0.95)	9.7 (6.1–15.5)	0.20 (0.09–0.46)	48 (18–131)
MRI	5	0.89 (0.86–0.91)	0.84 (0.69–0.93)	0.84 (0.77–0.90)	5.3 (3.7–7.7)	0.19 (0.09–0.38)	28 (13–61)
Comparison of diagnostic performances							
AI	6	0.93 (0.90–0.95)	0.83 (0.73–0.90)	0.90 (0.82–0.95)	8.2 (4.4–15.3)	0.19 (0.11–0.32)	43 (17–110)
Experienced radiologist	6	0.81 (0.78–0.85)	0.73 (0.65–0.79)	0.90 (0.83–0.95)	7.5 (4.2–13.4)	0.30 (0.23–0.40)	25 (12–52)

### 3.5 Subgroup analysis of different types of AI algorithms and image modalities

For the subgroup analysis of different types of AI algorithms, 10 studies used the ML algorithm and five studies used the DL algorithm. The pooled AUC, sensitivity, and specificity of the ML model for predicting LN metastases were 0.91 (95% CI 0.88–0.93), 0.84 (95% CI 0.74–0.90), and 0.87 (95% CI 0.82–0.90), respectively. The pooled AUC, sensitivity, and specificity of the DL model for predicting LN metastases were 0.92 (95% CI 0.89–0.94), 0.74 (95% CI 0.44–0.91), and 0.90 (95% CI 0.81–0.95), respectively. Our results showed that both ML and DL algorithms have the potential to predict the LN metastasis and no significant difference was found between the ML model and the DL model (**Supplementary Figures 1–4**).

For the subgroup analysis of different types of image modality, nine studies used CT features to train AI models, and the pooled AUC, sensitivity, and specificity for predicting LN metastases were 0.94 (95% CI 0.92–0.96), 0.81 (95% CI 0.62–0.92), and 0.92 (95% CI 0.87–0.95), respectively. Five studies used MRI features to train AI models, and the pooled AUC, sensitivity, and specificity for predicting LN metastases were 0.89 (95% CI 0.86–0.91), 0.84 (95% CI 0.69–0.93), and 0.84 (95% CI 0.77–0.90), respectively. The pooled results showed that the AI model based on CT has the potential to predict LN metastasis, and the pooled AUC of the AI model based on MRI for predicting LN metastases in OSCC was significantly lower than that of the model based on CT (**Supplementary Figures 5–8**).

The outcomes of the subgroup analysis are shown in **Table 2**; **Supplementary Figures**.

### 3.6 Comparison of diagnostic performances with experienced radiologists

Six studies with sufficient data compared the diagnostic performance of AI models and experienced radiologists. The pooled AUC, sensitivity, and specificity of the AI model for predicting LN metastases were 0.93 (95% CI 0.90–0.95), 0.83 (95% CI 0.73–0.90), and 0.90 (95% CI 0.82–0.95), respectively. For the experienced radiologists, the pooled AUC, sensitivity, and specificity for predicting LN metastases were 0.81 (95% CI 0.78–0.85), 0.73 (95% CI 0.65–0.79), and 0.90 (95% CI 0.83–0.95), respectively. The pooled results showed that experienced radiologists have a good ability to predict LN metastasis, but the pooled AUC for predicting LN metastases was significantly lower than that of the AI model (**Supplementary Figures 9–12**).

The comparison outcomes are shown in **Table 2**; **Supplementary Figures**.

## 4 Discussion

To the best of our knowledge, this is the first meta-analysis to quantitatively assess the diagnostic value of AI in the prediction of LN metastasis in patients with OSCC. The findings showed that AI based on CT and MRI images performed extremely well in predicting LN metastasis in these patients. The respective pooled sensitivity and specificity values of 0.79 and 0.90, the AUC value of 0.92, and the practical values in the Fagan nomogram indicated the potential for using AI models in clinical practice.

At present, the surgical strategy for OSCC with LN metastasis (LNM) includes both radical resection of the primary tumor and dissection of the neck LNs to varying degrees (37). Unnecessary surgical dissection of non-metastatic LNs can lead to postoperative complications, while delayed dissection of metastatic LNs can result in tumor progression. Therefore, the accurate evaluation of LN status is closely associated with the prediction of prognosis and the choice of surgical strategy. Although ultrasound-guided fine-needle aspiration is now performed for determining LN status with high specificity, the method is invasive and has major drawbacks such as reliance on the skill and experience of the surgeon, sampling error, and post-biopsy complications (38–40). CT and MRI are widely used non-invasive tools for diagnosing LN metastasis in patients with OSCC. However, even when interpreted by experienced radiologists, both imaging modalities can show poor efficacy in the diagnosis of LNM, with sensitivity and specificity values of CT between 67%–77% and 68%–72%, respectively, while the sensitivity and specificity values of MRI were 0.66% and 0.68%, respectively (10, 41).

In recent years, medical imaging-based AI techniques have been developed and deployed in clinics. AI technology is based on large-scale data training and deep learning algorithms, which can extract features from medical images and perform accurate analysis and judgment (42). Radiomics is one of the hand-crafted feature-based models that allow high-throughput mining of quantitative image features in medical imaging, which are then used as inputs for ML models that are trained to classify patients in ways that can support clinical decision-making (14). DL represents a fundamentally different paradigm to ML and can automatically extract the higher-level features from medical images without human intervention, thus precisely preserving both the objectivity and nature of the data, and has achieved an outstanding performance in various medical tasks (43, 44). Compared to conventional diagnostic methods, diagnostic models using AI algorithms have the advantages of reproducibility, objectivity, and immediacy. Currently, ML and DL models based on medical imaging are being actively evaluated for the determination of LN status and are showing great potential (45–47).

Our findings revealed that AI algorithms exhibited commendable performance with an AUC of 0.92, significantly surpassing the performance of established conventional diagnostic imaging methods. This is better than the performance of experienced radiologists reported in six of the included studies (AUC of 0.81). This may be associated with the faster image-processing rates, the ability to work continuously, and the recognition of certain imaging features that could not be detected by radiologists. Considering the real-life clinical adaptation of using both radiologists and AI models, an AI–radiologist combination would drive developments in the AI field and reduce the burden on the healthcare system. For example, AI may be considered a virtual assistant that can assist the radiologist in increasing both productivity and diagnostic accuracy, which would be particularly useful for less-experienced radiologists.

ML is a branch of AI and DL is a subset of ML. In contrast to ML, DL learns directly by navigating the data space without the need for explicit feature predefinition or selection (14). Given the growing number of applications of AI in medical imaging, several studies have compared DL algorithms with those of ML and have reported substantial improvements in the performance with DL (48, 49). In this meta-analysis, most of the included studies employed ML (*n* = 10), with only five studies using DL (one study used both AI algorithms). The pooled results showed that both ML and DL algorithms have a good ability to predict LN metastasis, and no significant difference was found between the two algorithms. It is undeniable that DL algorithms based on automatic segmentation can significantly reduce the time spent on manual labeling. However, the requirement for more data is a limiting factor due to its being more prone to overfitting. Chen et al. (33) reported that a DL–ML fusion model improved the preoperative identification of LN metastasis in OSCC and outperformed other single-algorithm models. Thus, fusion modeling may also be a trend for AI applications, allowing AI to meet the stringent requirements for clinical utility in the future.

CT and MRI are effective tools for the diagnosis of LN metastasis in patients with OSCC. In the context of AI, it is not clear whether there is a difference between CT and MRI in diagnosing LN metastasis. In our subgroup analysis, we found that CT-based AI models had a higher pooled AUC than the MRI-based models [0.94 (95% CI 0.92–0.96) vs. 0.89 (95% CI 0.86–0.91)], and there was no significant difference in terms of sensitivity and specificity, possibly because AI can extract more imaging features about LN metastasis from CT. It is also possible that AI models with better predictive performance may be developed in the future based on CT. Nevertheless, MRI is better able to accurately evaluate submucosal diffusion and invasion of adjacent structures while defining the LN status due to its high resolution of soft tissue (50, 51). Therefore, the selection of optimal imaging tools requires a comprehensive consideration of patient characteristics, cost-effectiveness, and clinical needs.

There were several limitations in this meta-analysis. First, because all included studies were retrospective in nature, potential bias, such as case selection, could not be fully eliminated from the analysis. Second, varying degrees of heterogeneity were present in both the pooled analysis and subgroup analysis. It is assumed that the high heterogeneity can be attributed to variations in the different patient populations, scanner technology, sample sizes, and targeted areas. Thus, the summary estimate values must be interpreted with caution. Third, most of the included studies were performed in Asia, and there is thus a potential for geographical bias. Last, few studies have conducted external tests to verify the performance of the AI models. Due to the unique characteristics of patients in different institutions, AI models developed using data from a single institution are usually limited in their broader implementation. Hence, high-quality multicenter prospective studies are needed to overcome biases in AI implementation and verify the prediction performance of AI models in the future.

In summary, the results of the analysis suggested that the use of AI based on CT and MRI for predicting LN metastasis in patients with OSCC has significant potential. More high-quality AI research under more stringent benchmarks is needed for clinical practice in the future.

## Data availability statement

The original contributions presented in the study are included in the article/**Supplementary Material**. Further inquiries can be directed to the corresponding author.

## Author contributions

CD: Data curation, Formal analysis, Visualization, Writing – original draft. JH: Data curation, Formal analysis, Writing – original draft. PT: Visualization, Writing – review & editing. TX: Data curation, Writing – review & editing. LH: Visualization, Writing – review & editing. ZZ: Writing – review & editing. JS: Conceptualization, Methodology, Writing – review & editing.
