# Supplementary material for: Application of CT and MRI images based on artificial intelligence to predict lymph node metastases in patients with oral squamous cell carcinoma: a subgroup meta-analysis
Source: Front Oncol. 2024 Jun 18;14:1395159. doi: 10.3389/fonc.2024.1395159 (PMC11217320; doi:10.3389/fonc.2024.1395159)
Supplement: Supplementary file 1 [file Table_1.docx]

**supplementary table 1 “Search strategy”.**

| **Sources** | **Search in** | **MeSH terms** | **Limits** | **Search results** |
| --- | --- | --- | --- | --- |
| Cochrane Library | Search manager | ("Artificial intelligence" OR "deep learning" OR "convolutional neural network" OR "machine learning" OR "automatic detection" OR "radiomics" OR "radiomic") AND ("CT" OR "MRI") AND ("Lymph node" OR "lymph node metastasis") AND (" oral squamous cell carcinoma OR "oral tongue squamous cell carcinoma” OR “head and neck squamous cell carcinoma”) | None | 0 |
| PubMed, (MEDLINE) | N/A | ("Artificial intelligence" OR "deep learning" OR "convolutional neural network" OR "machine learning" OR "automatic detection" OR "radiomics" OR "radiomic") AND (“computed tomography scan” OR “CT” OR “computed tomography” OR MRI OR “magnetic resonance imaging”) AND ("Lymph node" OR "lymph node metastasis") AND (“oral squamous cell carcinoma” OR “OSCC” OR “oral tongue squamous cell carcinoma” OR “OTSCC” OR “Head and Neck Neoplasms” OR “head and neck cancer” OR “HNC” OR “HNSCC” OR “head and neck squamous cell carcinoma" ) | Research articles, years (2010-2024) | 52 |
| EMBASE | Quick search | ('artificial intelligence'/exp OR 'artificial intelligence' OR 'deep learning'/exp OR 'deep learning' OR 'convolutional neural network'/exp OR 'convolutional neural network' OR 'machine learning'/exp OR 'machine learning' OR 'automatic detection' OR 'radiomics'/exp OR 'radiomics' OR 'radiomic') AND ('ct'/exp OR 'ct' OR 'mri'/exp OR 'mri') AND ('lymph node'/exp OR 'lymph node' OR 'lymph node metastasis'/exp OR 'lymph node metastasis') AND ('oral squamous cell carcinoma /exp' OR 'oral squamous cell carcinoma' OR 'oral tongue squamous cell carcinoma'/exp OR 'oral tongue squamous cell carcinoma' OR 'head and neck squamous cell carcinoma'/exp OR 'head and neck squamous cell carcinoma') | None | 113 |
| Web of Science | N/A | (Artificial intelligence OR deep learning OR convolutional neural network OR machine learning OR automatic detection OR radiomics OR radiomic) AND (CT OR MRI) AND (Lymph node OR lymph node metastasis) AND (oral squamous cell carcinoma OR oral tongue squamous cell carcinoma OR head and neck squamous cell carcinoma) | None | 58 |
