# Supplementary material for: Application of CT and MRI images based on artificial intelligence to predict lymph node metastases in patients with oral squamous cell carcinoma: a subgroup meta-analysis
Source: Front Oncol. 2024 Jun 18;14:1395159. doi: 10.3389/fonc.2024.1395159 (PMC11217320; doi:10.3389/fonc.2024.1395159)
Supplement: Supplementary file 2 [file DataSheet_1.docx]

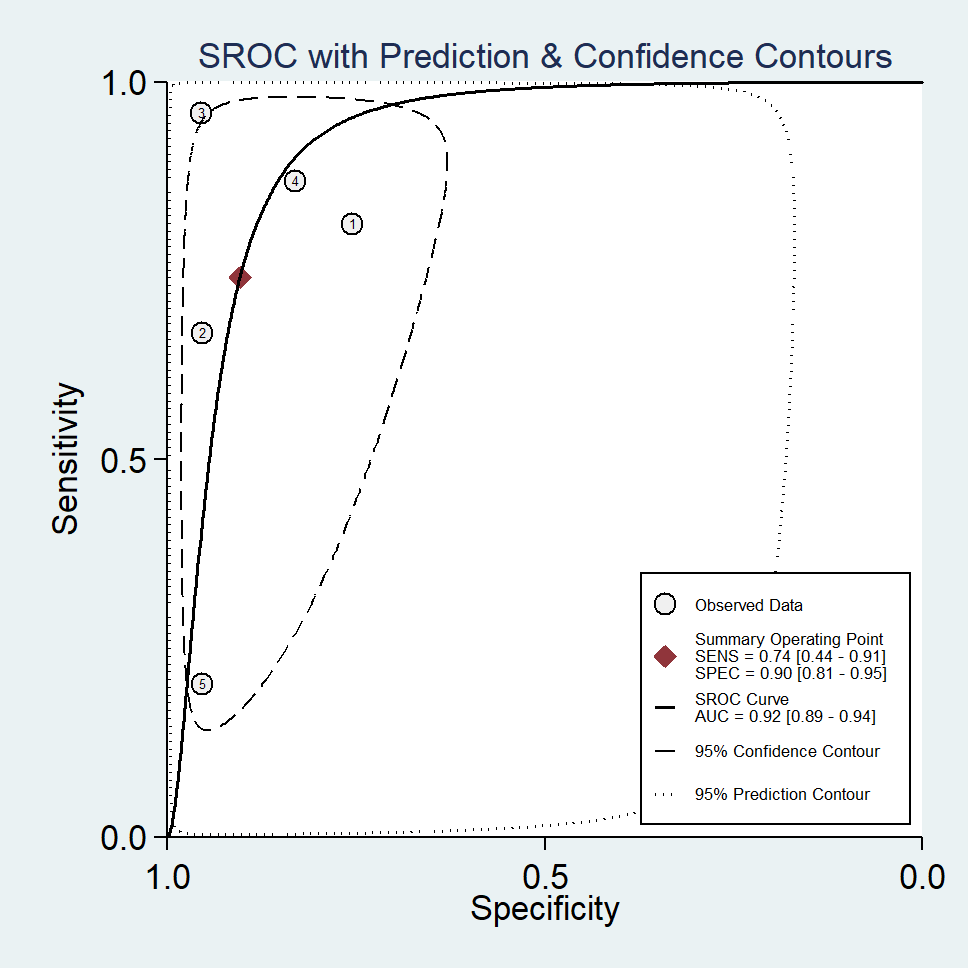


Fig. 1 SROC curve of DL


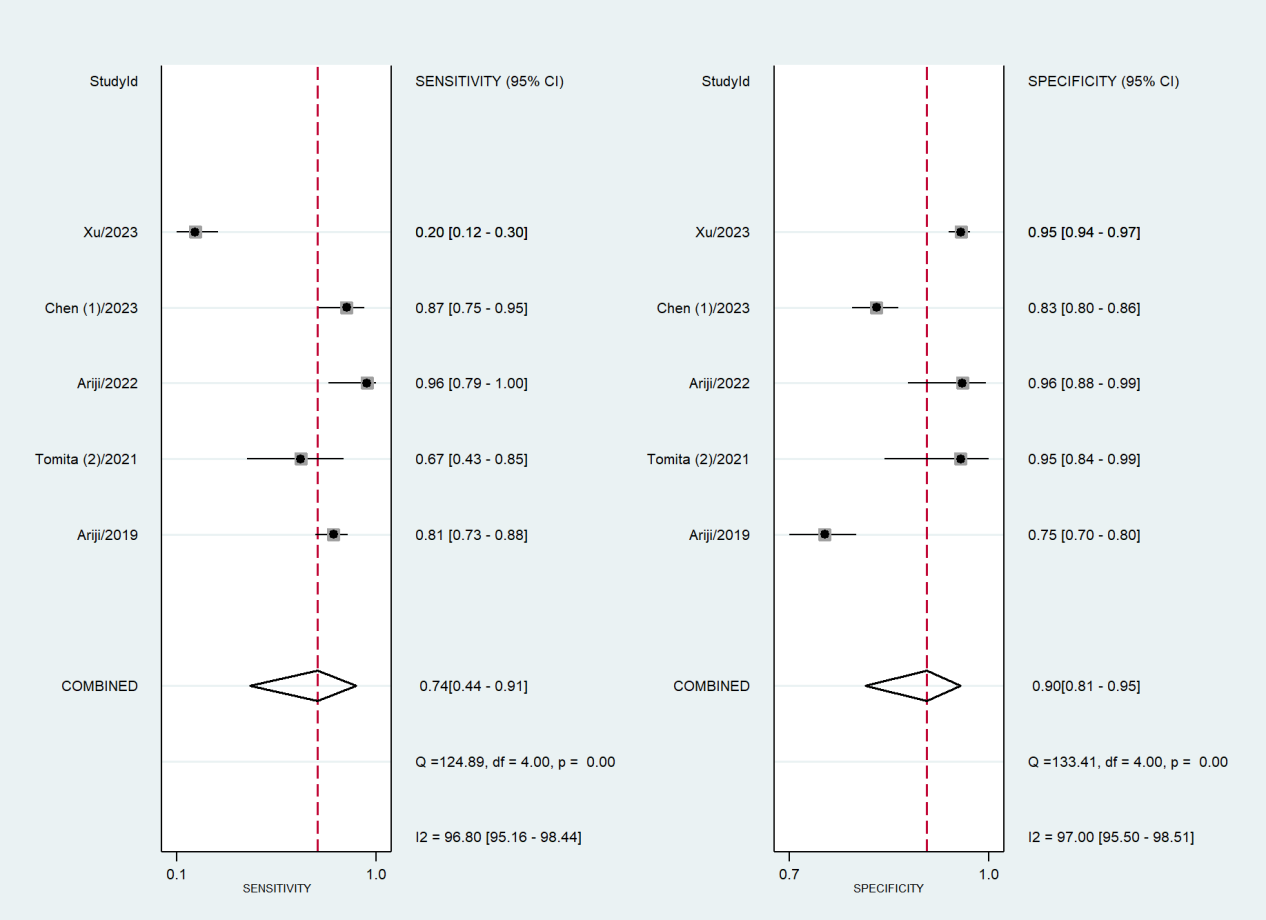


Fig. 2 forest plots of DL


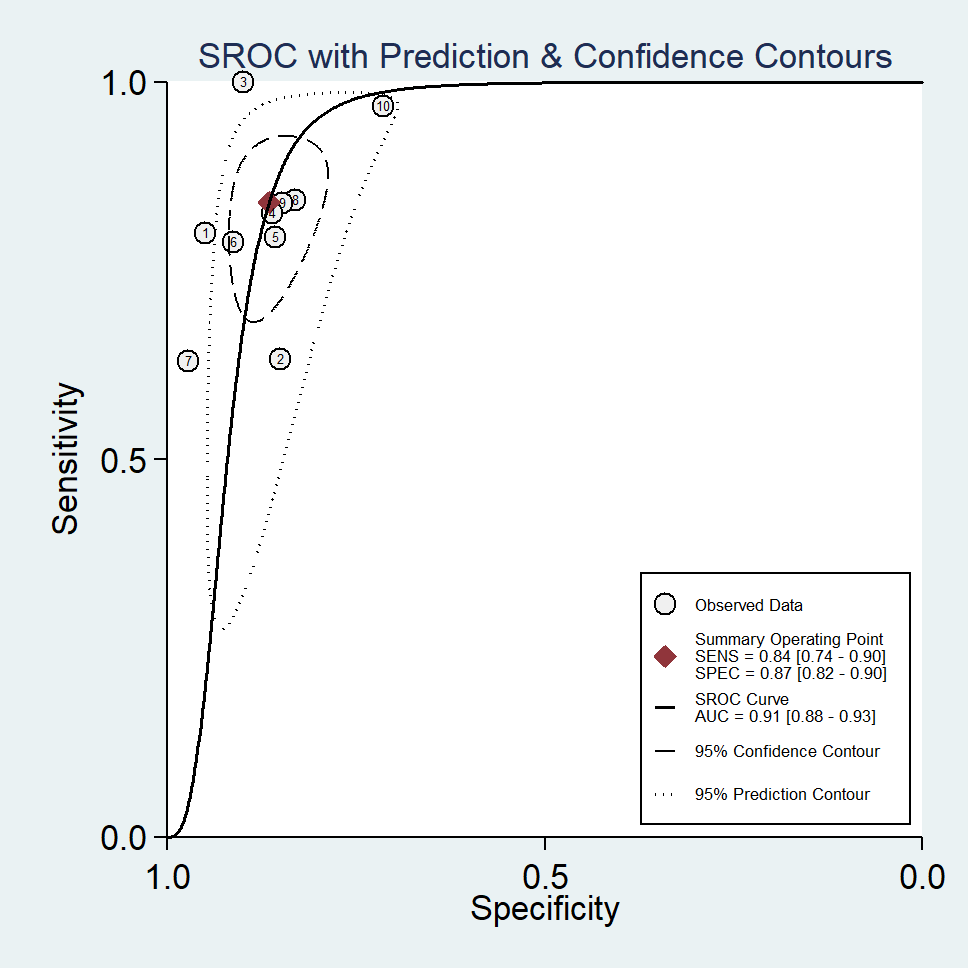


Fig. 3 SROC curve of ML


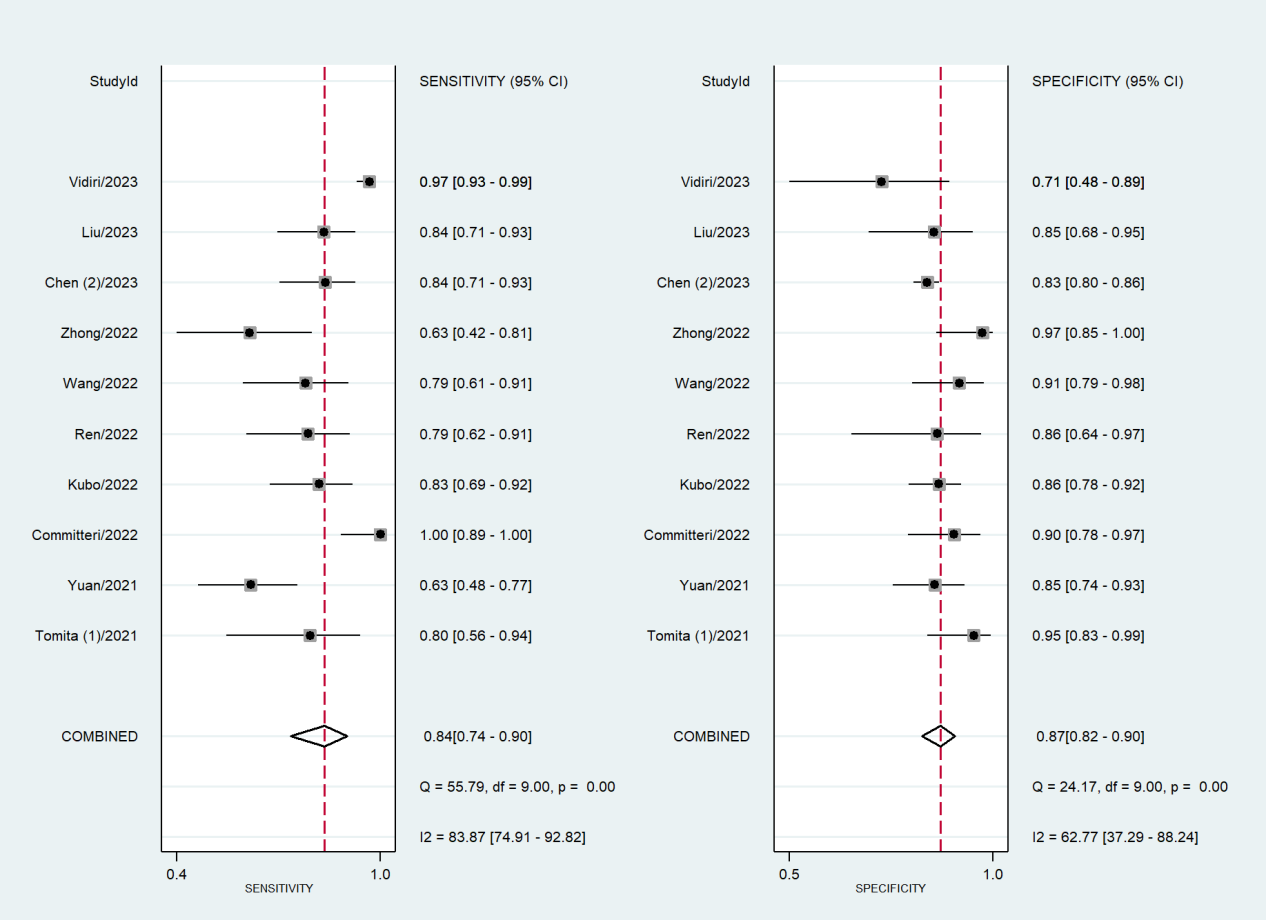


Fig. 4 forest plots of ML


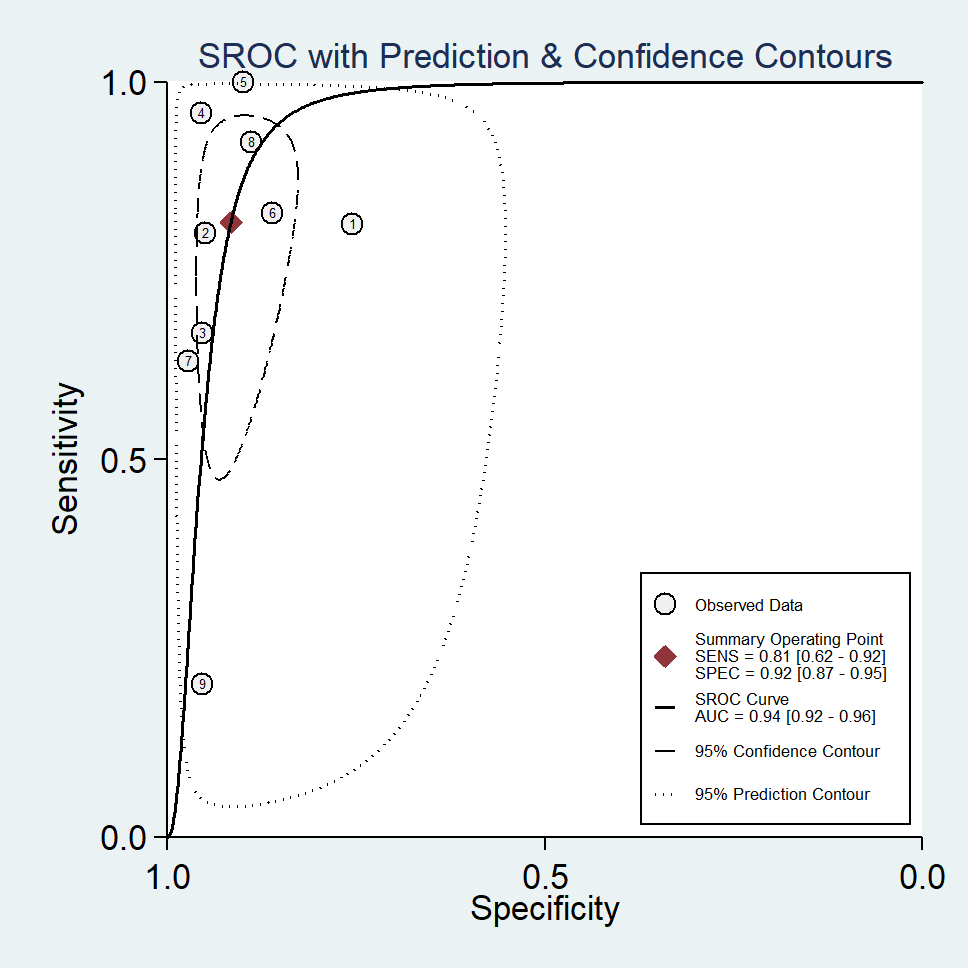


Fig. 5 SROC curve of CT


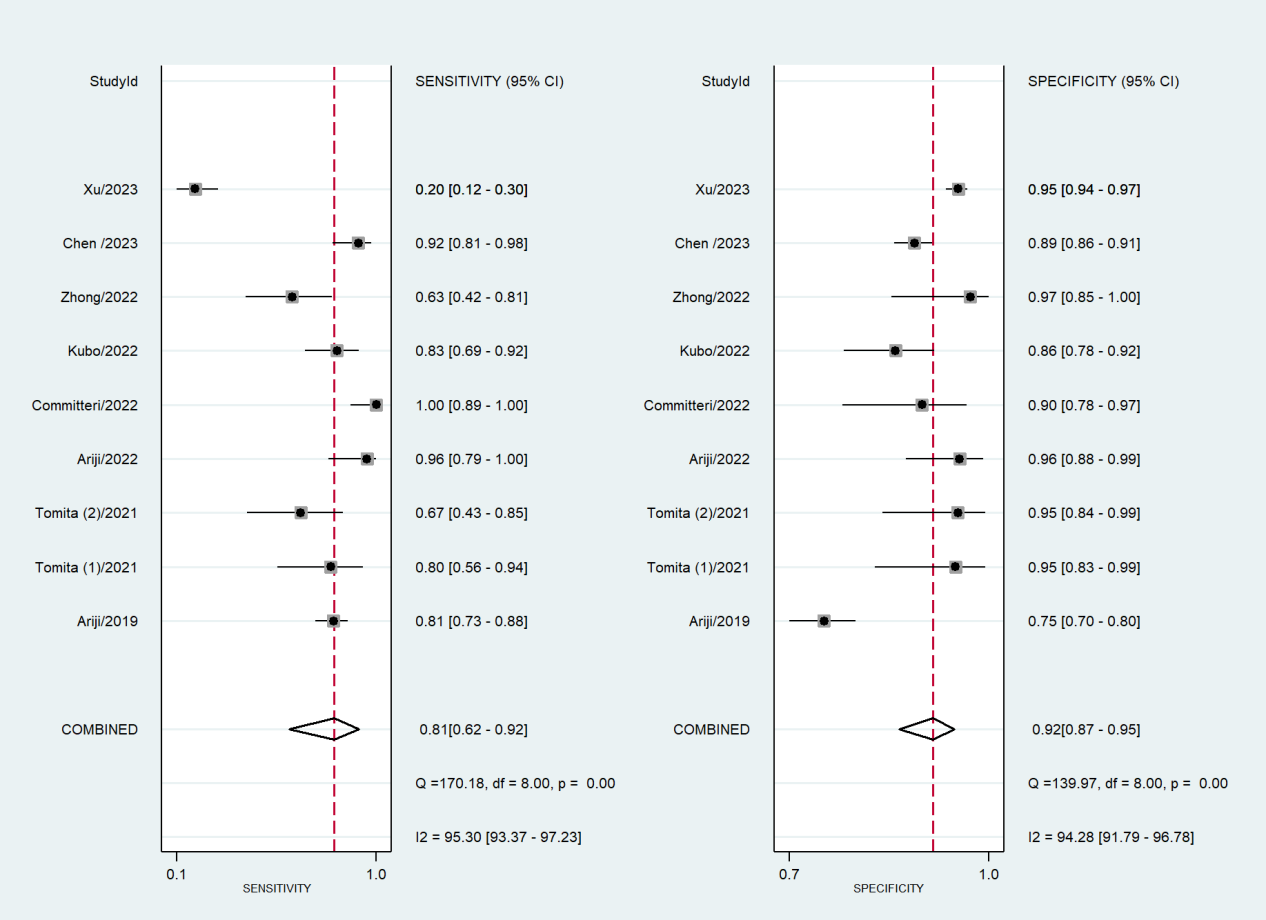


Fig. 6 forest plots of CT


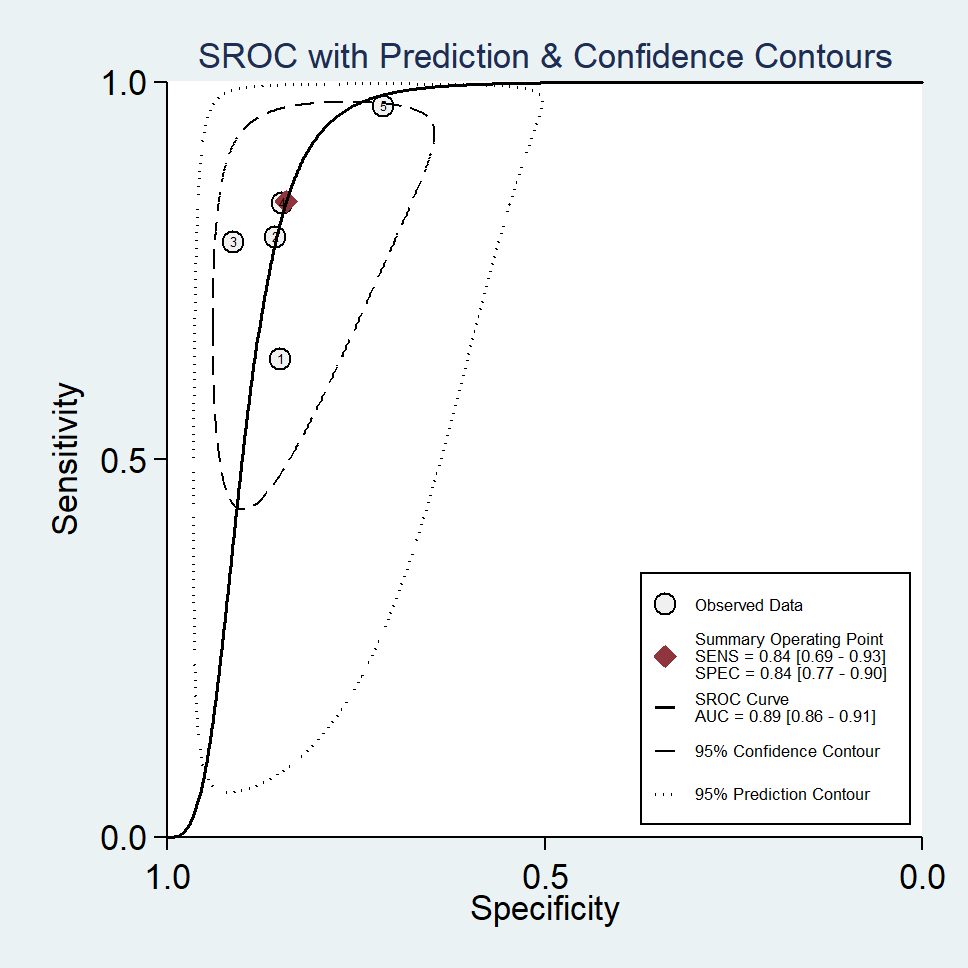


Fig. 7 SROC curve of MRI


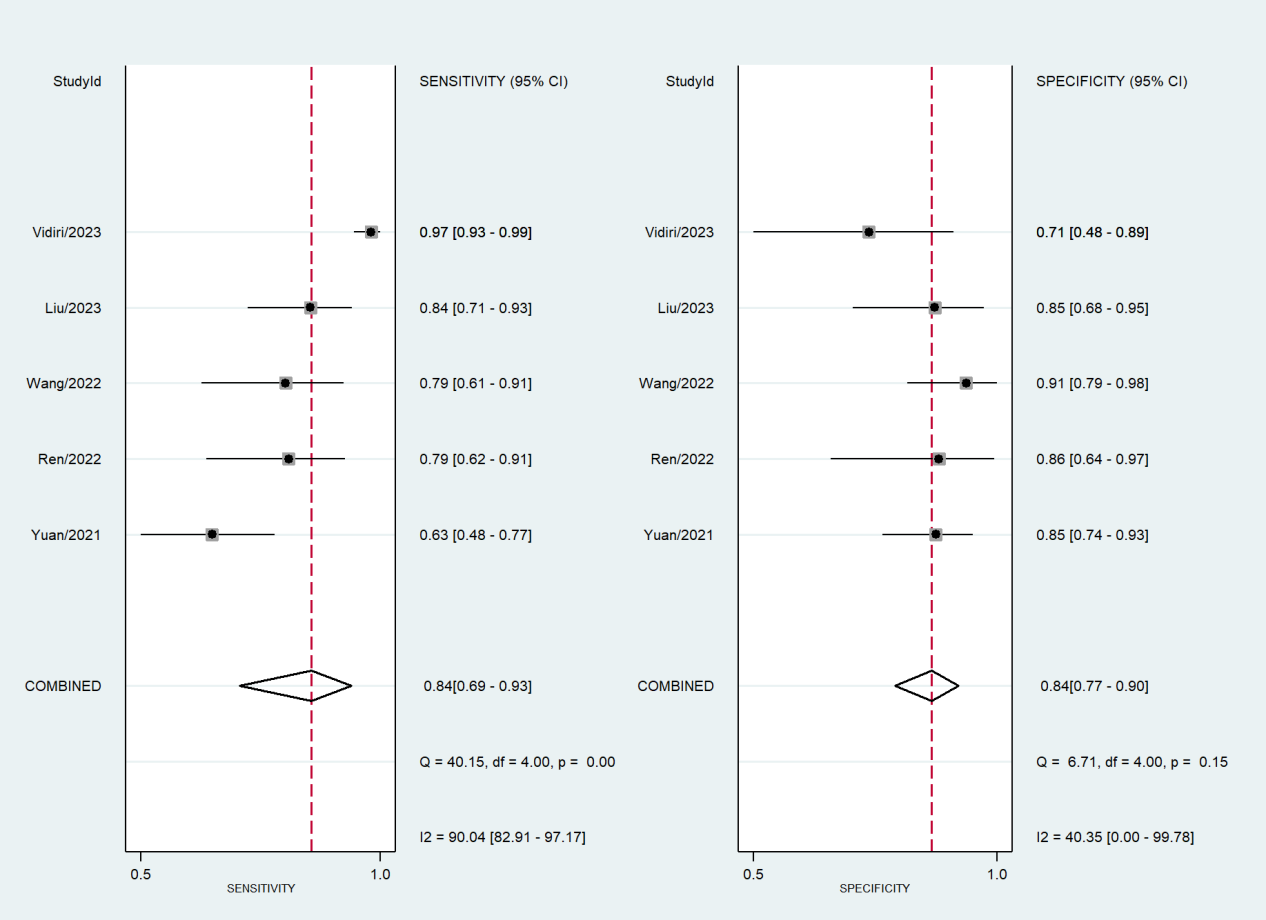


Fig. 8 forest plots of MRI


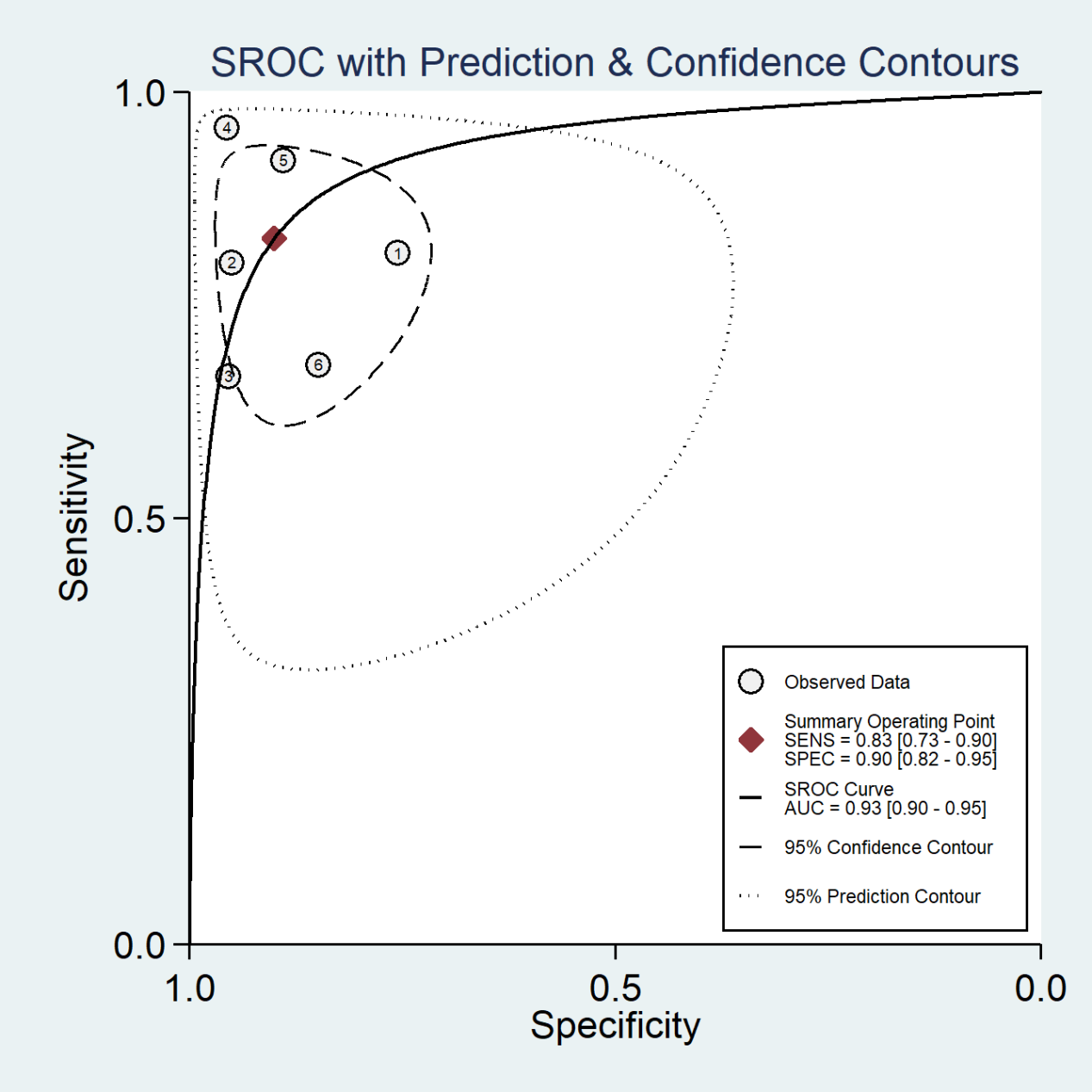


Fig. 9 comparsion SROC curve of AI


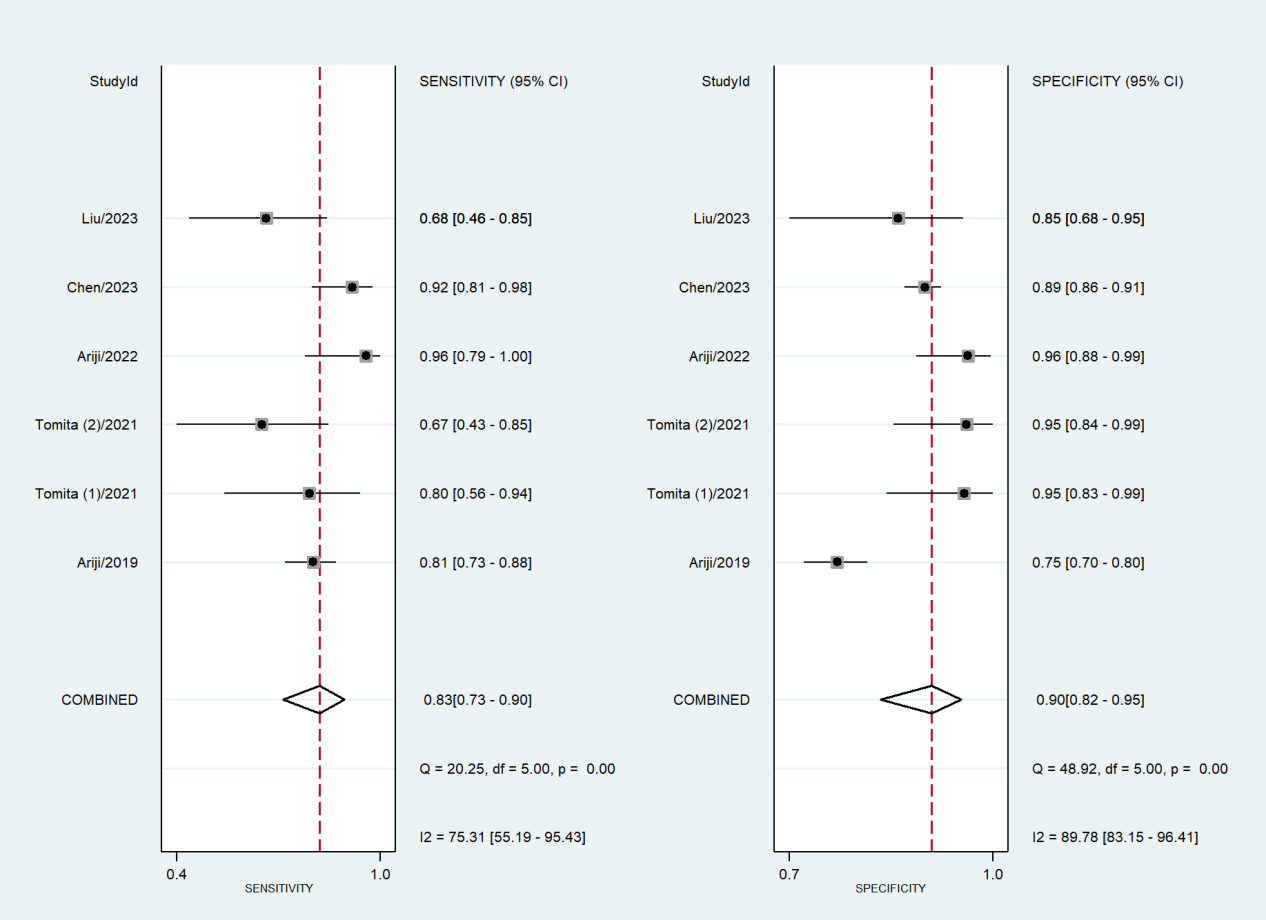


Fig. 10 comparsion forest plots of AI


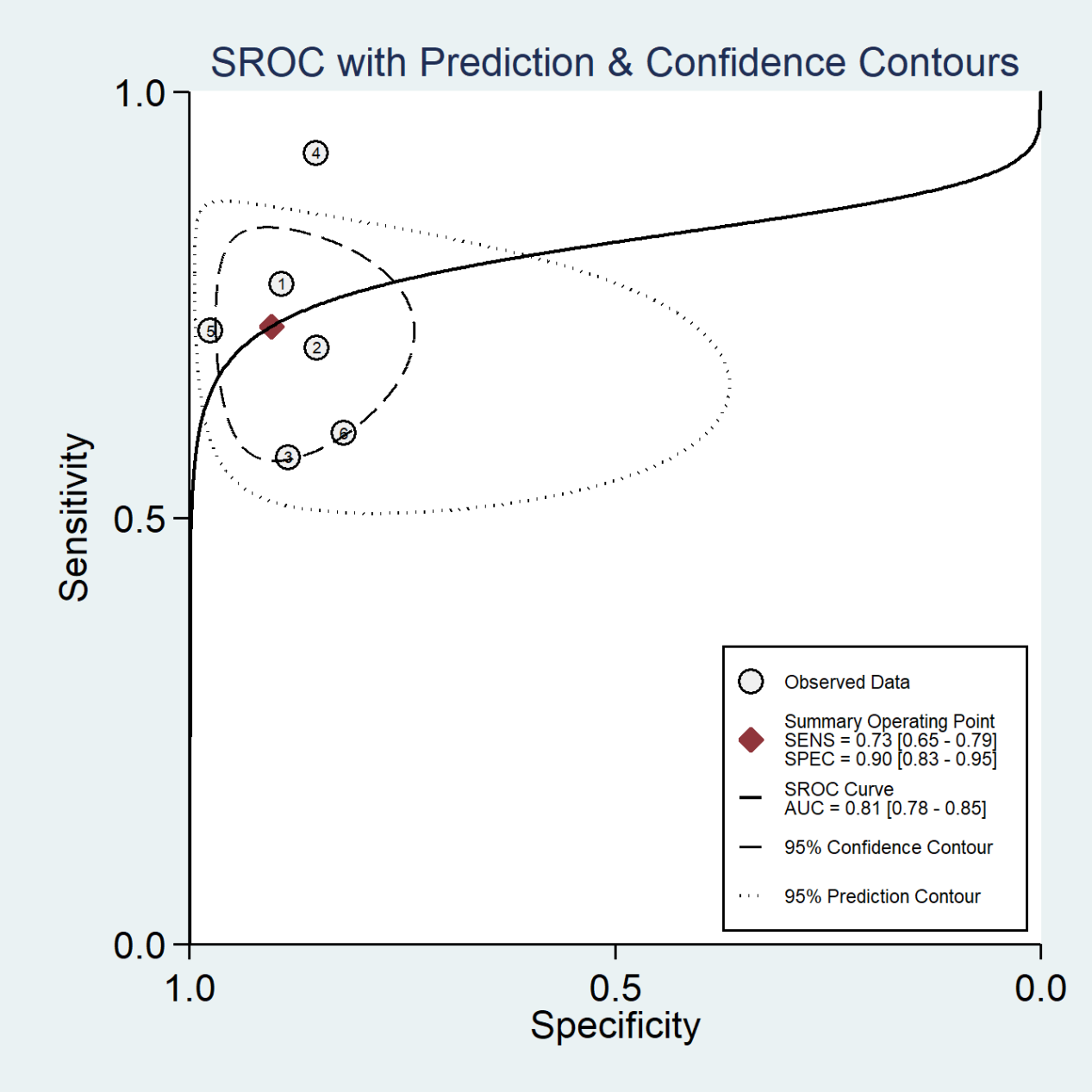


Fig. 11 SROC curve of experienced radiologist


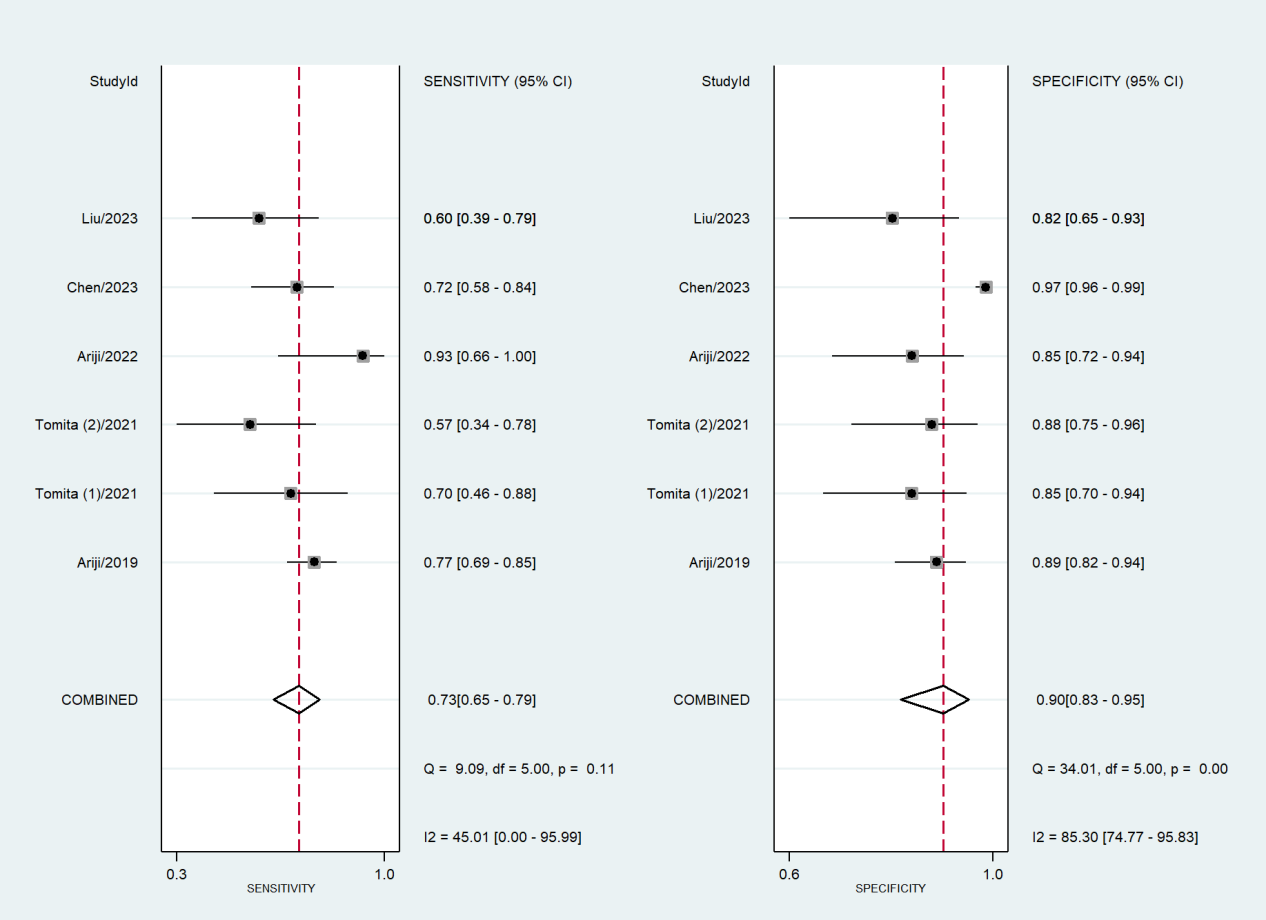


Fig. 12 forest plots of experienced radiologist
